# A Course of Clinical Lectures, Delivered at the Hôtel Dieu, Paris, for the Session 1842-’43

**Published:** 1843-04-29

**Authors:** A. F. Chomel

**Affiliations:** Paris


### THE MEDICAL EXAMINER,

### And Retrospect of the Medical Sciences.

Vol. VI.] PHILADELPHIA, SATURDAY, APRIL 29, 1843. [No. 8.

### A COURSE OF CLINICAL LECTURES,

*Delivered at the Hôtel Dieu, Paris, for the Session* 1842–'43.

BY A. F. CHOMEL, M. D.

### LECTURE VI.

### SCIRRHOUS DEGENERATION OF THE LIVER.

At No. 9 of the Salle Saint-Agnes, commenced Dr. Chomel, is a patient who entered the hospital with considerable effusion into the abdomen, and with a tumor in the hepatic region, of such volume as to occupy also the two hypochondriac regions. This tumor extends beyond the false ribs of the right side by four fingers breadth, and is then directed obliquely from left to right in the direction of the anterior or sharp edge of the liver; it is evidently formed by the liver itself, which appears to be profoundly altered. It might be thought that the tumor is formed by the epiploon; but a tumor thus formed would occupy chiefly the middle of the abdomen, whereas this tumor is much more developed on the right side than on the left. We have, then, in this case, a considerable ascitic effusion, with an abdominal tumor, recognizable by palpation, exercised with the precautions necessary when a liquid is interposed between the viscera and the abdominal parietes;* and this tumor is manifestly formed by the liver. The patient, however, does not present any symptom of icterus; the skin is pale, and somewhat florid, without any appearance of that yellow tint which is one of the characters of organic affections of the abdomen. This is a fact which contrasts remarkably with the presumed nature of the disease.

On his entrance, three days since, this patient had still some appetite; the tongue was white; there was no thirst; if he eat a little, he immediately experienced inconvenience, and then pains in the stomach, which obliged him to observe a severe abstinence. His stools were liquid and yellow; he had no fever.

To-day the same state continues; it would seem as if the liver were a little less voluminous than it was on the day the patient entered the hospital; this is probably due to that organ having somewhat changed its place, and thus presenting a smaller surface to the touch. As to the general condition, it would seem, on the contrary, to be aggravated; the ascites increases daily, and the difficulty of respiration has augmented in proportion.

Before discussing the nature of the disease in question, it will not be useless, in this place, to recall the antecedent history of the patient, such as we have collected it from himself. He has assured us that his health was habitually good; and that with the exception of slight colds, of which he cured himself very speedily, he never had suffered from any disease before the present attack. He was taken sick towards the latter part of December; at this period he perceived that his abdomen was somewhat enlarged, and a little white: afterwards his legs began to swell. Thus, from the declaration of the patient, we find that the disease is recent, and that its progress has been remarkably rapid. He told us, moreover, that he never had received a blow, or had a fall upon the region of the liver; he never has had any trouble, or experienced any reverses. Hence we can find neither a physical nor a moral cause for the development of the disease.

There remains another question to be proposed, after that of etiology, which is, as you see, involved in some obscurity; it is to know what is the exact lesion of the liver which gives rise to the morbid phenomena we observe; as to the seat of the disease there is no doubt, for the ascites is evidently only a consecutive state, or symptom of the affection of the liver.

What is, then, the nature of the hepatic lesion? What is it which produces the anormal development which this organ presents? This augmentation of volume may depend on several conditions, whose characters we should investigate here. It can be produced by an inflammation of the liver, by a cyst, by an abscess, a scirrhous degeneration, or by a simple hypertrophy consecutive to a cirrhosis, which latter is, however, very rare. The rapidity in the march of the affection would lead us to believe in the existence of a phlegmasia of the hepatic tissue; but an acute inflammation of the liver would give rise to a febrile movement of more or less intensity. Now, in our patient the pulse has not been affected; hepatitis, too, is ordinarily accompanied with an alteration of the biliary secretion with icterus; here we see this symptom, too, is wanting. Finally, it is very rare, in the climate of Paris, to see cases of spontaneous hepatitis; these are almost always traumatic. We have seen that this patient has had neither a blow nor a fall upon the side; so that the absence of the characteristic symptoms on one side, and also of the causes by whose influence it is ordinarily developed on the other, lead us at once to dismiss the idea of this affection, whilst there is nothing, on the other hand, which authorises our admitting it. The same motives lead us to reject also the idea of an abscess; for although, strictly speaking, a purulent collection may occur in the liver, without its being preceded by any well-marked inflammation, yet, as a general rule, the formation of an abscess is the last stage of inflammation of the liver. Besides, whatever, may be the origin of the suppuration, it is always accompanied by fever; and here it is entirely absent. Is it cirrhosis? This is not an impossible supposition; for although, in general, hypertrophy is not connected with this alteration of the liver, yet we sometimes find hypertrophy and cirrhosis united in the same subject. I have had occasion to observe two cases of this kind in my service; and as the coincidence is very rare, I deposited the pieces in the Dupuytren Museum. The existence, then, of hypertrophy does

* See Medical Examiner, p. 62.

not positively exclude the possibility of cirrhosis; but cirrhosis is ordinarily accompanied, if not with decided jaundice, with a dusky yellow tint of the skin, sufficiently characteristic, which in our patient is altogether wanting.

Is it, then, a multilocular scirrhous tumor? This last supposition seems to me more probable than the others. Certainly it is not common to see scirrhous degenerations of the liver develope themselves in so rapid a manner; their march is generally more slow; but, nevertheless, we sometimes see them developed with very great rapidity, and arrive at their last period without any appreciable antecedent stage; and this, too, in persons who offer all the appearance of fine health. That which induces me, above all, to incline towards this opinion in the present case is, that it is very common in such cases to encounter neither icterus nor sensible alteration in the urine, or in the alvine evacuations, nor to find any marked disturbance in the digestive functions, except in the very last stage. These circumstance arise from the fact, that in the interpaces of the enucleated tumors of the liver, the hepatic tissue remains sound, and the biliary secretion is consequently performed as well, or nearly so, as in the normal state. When we examine with care the relations of these tumors with the hepatic ducts, we find that they are compressed by the tumors in a manner altogether mechanical; and if the biliary secretion does become deranged, and consecutively the digestive functions, it must be attributed alone to the mechanical pressure exercised on the excretory ducts of the liver, and not to any real alteration in the secretion itself; a fact which is fully demonstrated in all our autopsies in a most evident manner. It is thus that we explain the appearance of icterus in the course of a scirrhous affection of the liver.

Thus, the absence of icterus, which, with regard to the other alteration of the liver, is a mere negative sign, becomes nearly a positive sign in connection with the organic alteration we suppose to exist in the present case; icterus here being an exception, and in a measure entirely accidental. Finally, on exploring with care the most superficial portion of the liver at the epigastric region, I discovered inequalities on the surface; salient, prominent points, with depressions; this is nearly a certain diagnostic sign of the existence of scirrhus, whenever this character is fully revealed, which, however, it is not in the present case, owing to the intra-peritoneal liquid. Another circumstance which prevents our recognizing this character distinctly, is the excessive sensibility of the liver, and the difficulty of continuing exploration for any length of time, without occasioning great pain to the patient. This excessive sensibility would seem to be due to the special alteration, of which the liver is the seat; and it may also be due to a certain amount of peritonitis.

In addition to the symptoms already enumerated, you may remark a dilatation of the superficial veins of the abdomen; they are slightly varicose; now this happens whenever the abdomen is anormally distended. The liver is also pushed up into the cavity of the chest, which explains the diminution in the respiratory murmur on the right side, whilst on the left side it is perfectly natural. This circumstance, joined to the dulness of the whole inferior half of the right side of the thorax, is a new sign indicative of the volume of the liver.

It is hardly necessary to tell you that the prognosis of this affection is exceedingly grave. The oppression goes on increasing, and tends to aggravate more and more the position of the patient. What is to be done under these circumstances? What is the treatment to be employed? Whatever we do, it is more than doubtful that we do not obtain any satisfactory result. Under this conviction our means are limited to the administration of opiates, with the faint hope of bringing some relief to the patient; for even these palliative means will probably be without effect.

[On the seventh day after the entrance of the patient into the hospital he died, the oppression going on increasing till death.

The autopsy showed a condition of the liver constituting a very remarkable pathological specimen. This organ had acquired twice its natural volume; its tissue was hard, and the surface was covered with small enucleated tumors, about the size of hazel nuts; in some portions they were in clusters, so as to occupy the whole substance of the liver; discreet in others, the hepatic tissue remaining sound in their intervals, of a brownish red colour. In incising the organ in its entire width, the tissue presented the appearance of a layer of a grayish yellow colour, marbled, in which the natural substance of the liver could not be distinguished. Thus nearly the whole substance of the liver was invaded and replaced by these masses of degenerated tissue.

It is very remarkable, and very difficult to explain, why there were no symptoms of jaundice with such profound alterations; and how the biliary secretion went on till the last in nearly a regular manner.

The abdominal cavity contained only a small quantity of serum. The other viscera were all healthy.]

*Paris, January*, 1843.

